# Enacting desired change in the present: Explaining prefigurative collective action during Chile's 2019 social explosion

**DOI:** 10.1111/bjso.70133

**Published:** 2026-09-24

**Authors:** David Clarke, John Drury

**Affiliations:** ^1^ School of Psychology University of Sussex Brighton UK

**Keywords:** collective action, ESIM, prefigurative politics, protest, resistance, social movements

## Abstract

Many contemporary movements confront existing power structures while experimenting with alternative social relations—a dynamic often discussed as prefigurative politics. Yet social psychology has prioritized explaining why people participate over examining what unfolds through collective action. This study examines how 16 participants made sense of their participation in Chile's 2019 social explosion. Using reflexive thematic analysis of in‐depth interviews, informed by the elaborated social identity model, we examine whether aspects of their involvement were understood in prefigurative terms and, if so, whether this was rooted in prior commitments, or shaped through participation, or both. Our analysis developed four interrelated themes: discovering a shared struggle, organizing and relating differently, experiencing alternatives as feasible and desirable and, for most, understanding their involvement as partially enacting the change they sought—a process we term *prefigurative realization.* Although all had prior political experience, these accounts suggest that participation—not prior commitments alone—contributed to prefigurative understandings, as existing commitments were validated and reconfigured through collective practice. These findings support for social psychology to approach collective action as a potentially transformative practice, where action is not only a means of pursuing social change but a site where change may begin to take shape.

## INTRODUCTION

Contemporary movements are redefining what it means to act collectively. From feminist initiatives and mutual‐aid networks to climate camps and popular assemblies, protesters are resisting injustice while experimenting with alternative ways of living, relating and organizing (Sitrin, 2020). Such cases are increasingly discussed as forms of *prefigurative politics*, in which participants enact through their present practices the social relations they aspire to see in wider society (Monticelli, 2022). Yet, while research on collective action has expanded rapidly (Thomas & Osborne, 2022), most studies continue to prioritize explaining *why* people participate (Drury, 2026). As a result, comparatively little attention has been paid to the transformative dynamics that unfold *within* action itself—particularly when participation becomes a site of prefigurative experimentation.

Against this backdrop, the present study examines the 2019 Chilean *social explosion (estallido social)* 1—a mobilization that has been analysed through the lens of prefigurative politics (e.g. Woods, 2022)—to explore how participants made sense of their involvement in the uprising. Drawing on the elaborated social identity model (Drury & Reicher, 2009) and in‐depth interviews with 16 participants, we investigate whether aspects of their involvement were understood in prefigurative terms and, where they were, how those understandings related to prior commitments and experiences of participation. In doing so, the study challenges the predictor‐outcome orientation that predominates in mainstream collective action research by foregrounding participation as a site of psychological and social change.

### Prefigurative politics in an age of crisis

Contemporary collective action unfolds amid compounding ecological, economic and political crises (Yates et al., 2024). As institutional responses lag behind the scale and urgency of these challenges, many social movements rely on self‐organized infrastructures—assemblies, networks, encampments—to coordinate and sustain action, while experimenting with alternative ways of organizing and relating alongside or outside established political structures (Monticelli, 2022).

These developments are often discussed through the concept of *prefigurative politics*: collective action in which participants seek to enact, within their present practices, aspects of the social relations they aspire to see in wider society (Breines, 1989; Monticelli, 2022). Prefiguration places the organization of struggle itself within the political project, linking how participants coordinate, make decisions, provide care and relate to one another, to the forms of collective life they seek to create (Boggs, 1977; Yates, 2015).

The autonomous infrastructures and forms of collective experimentation associated with prefigurative politics draw on much longer traditions of radical and autonomous organizing. Over recent decades, these practices have been discussed in relation to movements ranging from the Zapatistas to Occupy and more recent mass uprisings, where confrontation with existing power structures has unfolded alongside horizontal decision‐making, participatory coordination and forms of collective care (Monticelli, 2022; Raekstad & Gradin, 2020; Sitrin, 2020). Yet the presence of practices associated with prefiguration does not, by itself, reveal how participants understand them: they may be adopted for practical or strategic reasons without being experienced as enacting desired social relations. This raises social psychological questions about how participants understand such practices and how such understandings relate to what they bring to collective action and what unfolds through participation.

### The social psychology of collective action

Over the past two decades, research on collective action has grown rapidly, with influential models primarily seeking to explain when and why individuals participate (Thomas & Osborne, 2022). Researchers have identified key psychological predictors of participation, most prominently in work drawing on the social identity model of collective action (SIMCA; Agostini & van Zomeren, 2021; van Zomeren et al., 2008). This work demonstrates how shared identification, perceived injustice, efficacy and moral beliefs shape willingness to engage in collective action. In response to critiques concerning Western‐centrism and concerns about rising repression worldwide, studies have increasingly examined participation in non‐democratic contexts (e.g. Ayanian et al., 2021; Ayanian & Tausch, 2016). Growing concern over the climate crisis has likewise prompted research into the psychological drivers of climate activism (Bamberg et al., 2015; Wallis & Loy, 2021) and the role of future‐oriented beliefs in motivating collective action (Fernando et al., 2018; Wenzel et al., 2024).

Together, this work provides a strong empirical base for explaining the antecedents of participation across contexts. Yet much of this literature positions collective action primarily as the behavioural outcome of prior identities, appraisals and beliefs. By contrast, a smaller body of research has examined what happens *through* participation, including changes in identities, social relationships and perceived capacities (Drury & Reicher, 2000; Louis et al., 2016; Vestergren et al., 2017). One influential framework within this body of work is the elaborated social identity model (ESIM; Drury & Reicher, 2000, 2009; Stott & Reicher, 1998; Vestergren et al., 2019). Drawing on crowd research, ESIM conceptualizes collective action as an unfolding process in which interaction with other groups, such as the police, can transform collective identity: who is included within the in‐group, what actions appear legitimate and how the struggle is understood (Drury & Reicher, 2000, 2009; Vestergren et al., 2019).

Where policing is experienced as indiscriminate and illegitimate, identity boundaries may broaden and forms of action previously considered unfeasible or inappropriate may become legitimized. A broader shared identity may generate expectations of mutual support, while its successful enactment in practice—collective self‐objectification—can provide participants with a tangible sense of their collective capacity (Drury & Reicher, 2009). Unlike approaches concerned primarily with why people participate, ESIM therefore directs attention to how participation may transform participants' sense of who they are, how they relate to one another, what forms of action they regard as legitimate, and the meaning they assign to their struggle. These features make ESIM particularly relevant to prefigurative collective action, where a central question is how participants understand their collective practices and whether those understandings change through participation.

### The social psychology of prefigurative collective action

Despite recent calls for greater psychological engagement (Clarke & Drury, 2025; Cornish et al., 2016; Trott, 2016; Vestergren et al., 2025), prefigurative collective action has received considerably less attention in social psychology than in anthropology (e.g. Graeber, 2013; Maeckelbergh, 2011) and sociology (e.g. Monticelli, 2022; Yates, 2015). This has left unresolved whether, how and through what psychological processes participants come to understand practices often labelled prefigurative as enactments of desired social relations in the present (Clarke & Drury, 2025).

A small number of studies have begun drawing on ESIM to examine processes relevant to prefigurative collective action, including how collective practices may come to be understood as enacting alternatives rather than merely pursuing external outcomes (e.g. Acar & Uluğ, 2016; Aguilera & Drury, 2025; Mao et al., 2021). Acar and Uluğ (2016), for example, showed how conflict with the police and solidarity among previously divided groups contributed to a broader shared identity and enabled participants to experience an occupation to save a public park as embodying a more inclusive form of social life. Mao et al. (2021), meanwhile, showed how the same mutual‐aid practices during the COVID‐19 pandemic acquired different meanings depending on participants' political identification: for some they remained forms of volunteering, while for others, they modelled an alternative to existing social relations. Aguilera and Drury (2025), more directly, traced how participants in the 2018 Nicaraguan uprising, many with limited prior political experience, came to view practices developed through struggle as effective and fulfilling ways of structuring collective life and as partial enactments of the society they sought.

Taken together, these studies challenge the assumption that prefigurative understandings necessarily derive from prior ideological commitments. They suggest that collective practices and some of the social psychological processes unfolding through participation may also contribute to the development of such understandings, although the nature of this contribution remains unclear. This question is particularly relevant in mass uprisings, where diverse participants, established political repertoires and novel forms of coordination intersect under conditions of rupture and uncertainty (della Porta, 2009; Tilly, 2004).

### The present study

The present study examines how 16 participants in the 2019 Chilean social explosion experienced and understood their participation in the revolt. Using reflexive thematic analysis of in‐depth interviews (Braun & Clarke, 2013, 2021), it focuses on participants' accounts of how they organized and related to one another over time in demonstrations and cabildos (neighbourhood assemblies). Drawing on ESIM's account of how collective action can develop through intergroup interaction, the analysis attends to how participants described interactions with authorities and other protesters, and how these related to collective identity, the legitimacy of action, intragroup relations, perceived collective capacity and the meanings they assigned to the struggle. Specifically, it asks: (a) whether participants understood aspects of their involvement in prefigurative terms—that is, as enactments of desired social relations in the present; (b) where such understandings were present, whether they were rooted in prior commitments, shaped through participation, or both; and (c) what psychological processes were evident in participants' accounts of these understandings.

### The Chilean ‘social explosion’

The 2019 Chilean social explosion began in October 2019 after a rise in public‐transport fares in Santiago. Student‐led fare evasions and early protests intensified over several days and were met with heavy repression, including extensive, often indiscriminate, use of tear gas and pellet shotguns (Amnesty International, 2020; Human Rights Watch, 2019). Rather than being suppressed, the mobilization expanded nationwide into mass marches, roadblocks and rioting—the largest wave of protest since the end of the Pinochet dictatorship in 1990 (Cox et al., 2024; Somma et al., 2021). What began as a fare dispute became a broader challenge to political legitimacy and social inequality (Woods, 2022).

Alongside street‐level protest, the uprising also featured forms of organizing described as prefigurative, most notably the rapid spread of self‐organized neighbourhood assemblies (*cabildos*), horizontal coordination and rejection of traditional party leadership (Ureta et al., 2021; Woods, 2022). These drew on earlier cycles of mobilization and longer standing traditions of community organizing, which contributed organizational capacities and shared protest repertoires (Hilbink & Salas Ramos, 2024; Woods, 2022). Although largely autonomous, the 2019 *cabildos* were not without precedent. In 2016, President Bachelet's participatory constitutional process included provincial and regional *cabildos* designed to gather citizens' views on a new constitution (Figueroa Rubio & Jordán Díaz, 2021; Hilbink & Salas Ramos, 2024). The presence of these forms of organizing does not itself reveal how participants understood them, as practices labelled prefigurative may have been enacted for varied reasons, including as tactical responses to unfolding events.

*Cabildos* operated as open forums for interpreting events and coordinating responses (Ureta et al., 2021), with discussions increasingly centred on reform of the constitution inherited from the dictatorship (Woods, 2022). In November 2019, political parties agreed to initiate another constitutional process, leading to a plebiscite in October 2020 in which a large majority voted to draft a new constitution (Heiss, 2021; Sazo, 2023). Protest and cabildo participation continued during the constitutional process and the COVID‐19 pandemic, amid widespread institutional scepticism. Two successive drafts were later rejected, leaving the 1980 constitution still in place (Hilbink & Salas Ramos, 2024). Across the mobilization, over 30 people died during the unrest, thousands were injured and over 400 suffered eye trauma, largely due to police use of pellet shotguns (Amnesty International, 2020, 2021).

## METHODS

### Participants and recruitment

Participants were recruited via a flyer circulated by a local academic contact to individuals known to have participated in demonstrations and cabildos during the 2019 Chilean social explosion. Interested individuals contacted the first author directly, with further participants recruited through snowball sampling.

Sixteen participants (7 women, 9 men) took part. All had substantial prior political experience before the 2019 mobilization, including involvement in student movements, the 2018 feminist protest wave and community or union organizing. Participants were recruited from multiple regions of Chile: 12 were based in the Santiago Metropolitan Region, with one each from northern, central, south‐central and southern Chile. The sample included university students, graduates, lecturers and individuals engaged in community‐based work. Most participants were in early to mid‐adulthood, with two over the age of 50.

### Interview procedure

Semi‐structured interviews were conducted in Spanish by the first author between October 2024 and January 2025. Participants received information about the study and provided informed consent. The interview schedule was organized around broad sections aligned with the study aims and informed by ESIM, but was used flexibly in response to participants' own framings of events and experiences.

Interviews began by exploring participants' views of the pre‐2019 political context and their prior political experience (e.g. ‘How did you view national Chilean politics before the protests began?’). The next section examined experiences of demonstrations and cabildos, including how practices were adopted, understood and evaluated (e.g. ‘Can you tell me a bit about your participation in the cabildos?’). A subsequent section explored views of state repression and relationships with other protesters, focusing on views of authorities, legitimacy and intragroup relations before and during the mobilization (e.g. ‘What were your attitudes to the government following police and army violence against protesters?’). A final section invited reflections on empowerment, political possibility and whether participation reshaped understandings of national politics and social change (e.g. ‘How did your participation in the demonstration or assemblies affect your sense of what national politics might be like?’). Prefiguration was not introduced as a term or framing, nor were participants asked whether their actions were prefigurative. Instead, these questions allowed participants to describe the meanings they assigned to their experiences in their own terms. The full schedule is available in the online Supporting Information. Interviews were audio‐recorded, transcribed verbatim and anonymized. Extracts reported in English were translated by the first author. This study received ethical approval from the University of Sussex Research Ethics Committee (Ref: ER/DAC29/7).

Conducted approximately 5 years after the uprising, the interviews were treated as retrospective accounts of how participants remembered and interpreted their involvement, rather than as real‐time records of psychological change. Data collection coincided with the fifth anniversary of the social explosion. The first author spent 5 weeks in Chile during October and November 2024, visiting locations associated with the mobilization, participating in a protest marking the anniversary, meeting informally with activists and community members and keeping extensive field notes documenting observations, conversations and questions arising during the fieldwork. These activities supported recruitment, helped situate participants' accounts within their lived historical context and informed the interpretation of the interview material.

### Data analysis

Transcripts were analysed using reflexive thematic analysis (Braun & Clarke, 2013, 2021), theoretically informed by ESIM and suited to examining how participants interpreted their involvement and narrated the meaning attached to their collective practices. The first author conducted the coding and led the analysis, using NVivo to organize and code the transcripts. Analysis began with repeated reading and coding to identify recurring features in participants' accounts of their involvement in demonstrations and cabildos. Codes were then compared across interviews and examined in relation to the temporal structure of participants' narratives. Particular attention was paid to contrasts between prior political involvement and experiences during the mobilization, and to whether understandings of collective practices were described as changing or remaining stable. Candidate themes were developed by grouping codes around patterns of shared meaning and refined through repeated movement between coded extracts, complete transcripts, the research questions and the developing analytic account.

ESIM guided attention to how participants described relations with authorities and other protesters, changes in collective identity, experiences of collective empowerment and the meanings they assigned to their participation, but did not serve as a predefined coding framework. Prefigurative understandings were identified in participants' interpretations, rather than inferred from organizational form alone. Practices such as cabildos, horizontal coordination and self‐organized protest were therefore not treated as prefigurative simply because they resembled forms discussed in the literature. Instead, accounts were treated as prefigurative where participants described aspects of their involvement as enacting desired social relations, practising the kind of politics they wanted, organizing in ways consistent with the change they sought or briefly living aspects of the society they wished to see. Accounts were treated as primarily strategic or outcome‐oriented where practices were framed mainly as means for coordinating protest, channelling demands, achieving institutional change or improving movement effectiveness, without connecting them to the enactment of desired social relations.

The first author discussed developing codes, translated extracts, candidate themes and analytic claims with the second author during the analysis and writing process. These discussions helped question and refine the analysis by clarifying theme boundaries, examining the relationship between extracts and analytic claims and considering how interpretations were supported across the dataset and integrated into the paper's broader analytic narrative.

### Positionality

The first author is a British‐Latin American doctoral researcher fluent in Spanish. He approached the study with regional and political familiarity, but as an outsider to the Chilean national context. His political sympathy with the uprising and prior work on prefigurative politics supported his understanding of the stakes of participants' accounts, while also creating risks of over‐identification, romanticization and interpreting the material too readily through the study's theoretical concerns. The second author's role in developing ESIM further shaped the theoretical perspective brought to the analysis. These influences were worked with reflexively through repeated engagement with full transcripts, divergent accounts, discussion of developing interpretations between the authors and careful checking of the relationship between extracts and analytic claims.

## ANALYSIS

Across the interviews, 13 of 16 participants described aspects of their participation in the social explosion in ways we interpret as prefigurative, while the remaining three focused primarily on strategic and outcome‐oriented concerns. Drawing from across these interviews, we develop an account of how participants came to understand aspects of their participation in prefigurative terms through the interplay between prior activist and community organizing experience and the unfolding dynamics of the uprising. Four interrelated themes were developed: (1) ‘Chile woke up’: discovering a shared struggle; (2) organizing and relating differently; (3) alternatives that felt feasible and desirable; and (4) prefigurative realization: living the change they sought.

### Theme 1: *Chile woke up:* Discovering a shared struggle

This theme explores how interviewees came to see themselves as part of a common *we* in opposition to the authorities, as pre‐existing grievances were intensified by police violence widely perceived as indiscriminate and illegitimate. It considers what this shared identity—symbolically crystallized in the chant *Chile woke up*—came to stand for.

#### From a politicized minority to an awakened people

Looking back on the period before the uprising, most interviewees described themselves as belonging to a politicized minority within a society perceived as depoliticized and fragmented. Although all had participated in political or community organizing, they frequently characterized the broader public as largely apathetic and disengaged. For example:

Before the revolt I felt I was living in a completely alienated country… one living in a kind of poverty hidden by debt (…) in an individualism that you could feel, see in every corner… I felt deeply disappointed in people, in my community (…) I was involved in human rights groups helping former political prisoners during the dictatorship, and I often felt miserable; I'd get on a bus, look around… no one talked to anyone, no one said anything… they could be assaulting someone right next to you, and people would just keep walking. (Katia, Iquique)

This interviewee depicts an individualistic and apathetic society, while conveying the frustration of being politically active with little support. Across interviews, earlier political efforts were remembered as meaningful yet unable to gain public support, contributing to a widespread sense of political fatigue. Interviewees linked this to the repeated criminalization of protest by media and political elites, who framed it as disorder or extremism. The social explosion was instead described as a decisive rupture—a moment when this delegitimising narrative no longer held:

(…) even though there were lots of images of violence [from protesters], they didn't seem to be provoking the same public outrage as before… there was, if maybe not total support, at least a sense of justification for the anger that existed at that moment, which led to more and more people in the streets by the hour (…) People who used to just go about their lives without questioning much were suddenly all voicing criticism of everything that had been happening. That was really powerful!. (Andres, Santiago)

While previous protests had been portrayed as a nuisance or criminal, even disruptive or violent acts were now understood by interviewees as socially justified in the eyes of others. Many linked this shift to the scale and indiscriminate nature of police violence at the start of the protests. As one interviewee explained, the visibility of repression exposed it as illegitimate violence against ‘its own people’:

I heard lots of gunshots… I saw people shot in the eyes; people bleeding; beatings (…) I didn't recognise my own institutions. I had never seen violence like that before… from the State against its own citizens (…) Everyone could see it; it was all being filmed, circulating on social media, so there was no way to hide it or justify it. It was the State assaulting its own people, and that changed the perspective of many. You couldn't say that they were criminals, because what you saw were ordinary people protesting (…) (Alejandra, Santiago)

Widespread perceptions of state violence as both illegitimate (disproportionate, unjust) and indiscriminate (anyone was at risk) reinforced interviewees' sense that the protests now held much broader public support. The slogan *Chile despertó* (‘Chile woke up’) symbolized this shift, capturing an awakening from apathy and the recognition that Chilean society itself was in the streets:

That's why the uprising was so powerful… it was about waking up. One of the main chants, the first great cry, was “Chile woke up”, and we chanted it through the streets. It was, at last, a response to that kind of slumber we'd been in… of saying, look, we see all this [injustice], we know the names, the dates, everything, and yet we do nothing. And suddenly a mass of people appeared, symbolically coming together around the idea that Chile had woken up. It was beautiful… that awakening was beautiful.(Paula, Santiago)

In this way, *Chile woke up* expressed a broadening of collective identity: interviewees no longer understood themselves as a politicized minority within an apathetic society, but as part of an awakened people united by shared grievances and opposition to the system the government represented.

#### Defining what the uprising stood for: Dignity and autonomy

Interviewees described the revolt as quickly expanding beyond the metro fare dispute to injustices accumulated over the three decades since the dictatorship, bringing diverse demands together in a shared struggle. As one interviewee explained:

There was a great deal of discontent. In fact, the main chant appeared quite early on: “*it's not 30 pesos, it's 30 years*.” So we took to the streets… mostly young people to fight against the government (…) and they behaved just like during the dictatorship, with the same brutality, and for many of us that violence was seen as part of something broader. And I think that's how this movement, which belonged to millions, became so multifaceted; there were social demands like education, health, pensions, but also feminist, cultural, and environmental ones. (Pablo, Punta Arenas)

This account illustrates how interviewees' protester identity extended beyond immediate opposition to the government, encompassing diverse social demands within a broader struggle for change. Within this framing, interviewees frequently invoked dignity to articulate what the uprising stood for, understanding it as being treated with respect and being able to live decently:

I mean, we wanted what's obvious… what any human being needs to live with dignity. It wasn't a left‐wing movement; we weren't asking for socialism or any ideological programme. What we were demanding was better living conditions, better treatment, greater dignity (…) We were confronting a structure that denied us our dignity. (Pablo, Punta Arenas)

Alongside dignity, autonomy featured prominently in how interviewees defined themselves, with the social explosion described as ‘self‐convened’: that is, organized independently of political parties or institutions. For example:

In the first few months of the uprising, everyone was asking who was behind this. President Piñera claimed it came from Venezuela… that it was all manipulated. Political parties tried to get involved, but their militants who went to the cabildos stayed quiet; they were embarrassed to say that they belonged to a party (…) They weren't the ones convening or giving direction to the protests or to the cabildos (…) It was all self‐convened, popular, broad in that sense. (Antonio, Santiago)

Here, autonomy is narrated as a defining feature of the revolt through rejection of external leadership and the affirmation of collective self‐organization.

### Theme 2: Organizing and relating differently

This theme explores how interviewees organized demonstrations and cabildos as spaces that contrasted with the hierarchical and individualized relations they opposed. They described autonomous forms of organization and relations grounded in dignity, community and equality—commitments associated with the emergent protester identity we described in Theme 1.

#### Autonomy in practice

When interviewees described their participation in the uprising, they all emphasized the effort to keep the social explosion independent of political parties and traditional social movements. In the decades prior, political decision‐making was largely channelled through parties and institutions that interviewees viewed as unrepresentative and ineffective, leaving them feeling like powerless spectators. For many, the uprising reversed this dynamic: instead of waiting for instructions from established political actors, demonstrations and cabildos were organized as autonomous, open and voluntary. For example:

The self‐convened spaces… protests, plazas, street corners… were profoundly democratic (…) Unlike the technocratic, efficiency‐oriented, and unrepresentative system we'd grown used to, here we were reclaiming our power as citizens, regaining the right to voice our opinions. Everyone came independently – without leaders, without organisations – to talk, to listen to each other, to take notes… we even tried to make speaking time equal for everyone (…) They were liberating spaces where you could voice what you felt and thought, and let it out openly in public. (Antonio, Santiago)

As this interviewee stresses, rather than being directed by formal organizations, those present decided for themselves when to meet, where to protest and what to deliberate on. By organizing without mediation, they experienced autonomy as a collective and liberating practice, temporarily reversing decades of hierarchical and exclusionary politics.

#### Dignity in practice

Interviewees described gathering in the streets and assemblies as a reversal of everyday indignities experienced before the uprising. After years of feeling overlooked, silenced and isolated, they described standing together, reconnecting with fellow citizens and refusing to disperse even under threat of repression as profound moments of collective defiance and dignity. For example:

We kept taking to the streets [despite curfews] because it was what had to be done; because we couldn't stay silent any longer. We felt a need to make ourselves heard, to say “enough”. It was like a collective dignity – the dignity of saying “no more”… of being there together even if they fired tear gas, even when they repressed you. (Carolina, Santiago)

As the extract conveys, for many, dignity in the uprising was not an abstract value but something enacted through collective action and refusal. By standing together despite repression, interviewees experienced the streets as a site where dignity was both practised and reclaimed.

#### Community in practice

Many interviewees described how the insistence on autonomy and dignity as defining principles of the revolt shaped the ways they related to one another. Contrasting the fragmentation of everyday life under neoliberal policies, they spoke of the joy of feeling part of an awakened people. They recalled how communal relations became central to the experience of protest:

During the protests, I had the feeling that everyone was there, that we made up a people… a more unified, more equal country (…) we would sing together, dance together, celebrate together, and care for each other (…) There was this feeling of being a nation, a people, that were really building something together, and that it wasn't coming from above… it was us building it. It wasn't the state telling us: “This is Chile, you were born here, your ID says you're Chilean”. It was something else… a lived, spontaneous sense of unity, a real community. We didn't know exactly what we were building; we only knew that we were there, all of us, working together for something in common, all of us as citizens… (Andres, Santiago)

In this way, dancing and inhabiting public space momentarily dissolved hierarchies and made tangible forms of coexistence structured on their own terms rather than those of the powerful. In a society long constrained by isolation, communal relations reflected the stance they had taken: if no party or institution could speak for them, they had to organize themselves; if every person's worth mattered, then solidarity and care towards one another became the only appropriate way to relate.

#### Horizontality and participatory decision‐making in practice

Many interviewees emphasized that protests and cabildos came to be organized through horizontal and participatory forms of decision‐making, reflecting commitments to autonomy and dignity. Thus, they recounted how decisions were made collectively rather than through formal leadership structures:

In the neighbourhood assemblies, decisions were made collectively – everything was decided by everyone. There were no hierarchies, no one in charge. Each working group had its own role, and then we'd come together to share the agreements. If one person coordinated one week, someone else would take over the next. There was constant rotation because we didn't want anyone to hold on to power. It was really impressive seeing people who may never have participated in this kind of thing… speaking up, voting, coordinating. In those spaces, we experienced a different kind of politics, unlike party politics… (Mario, Santiago)

As the extract suggests, since there was a commitment to allow people to make decisions autonomously and to ensure that each person's contribution was recognized, everyone had to be listened to. Through practices such as turn‐taking, small‐group work and parity, interviewees experienced equality and participatory democracy as tangible social arrangements rather than abstract ideals.

Taken together, these accounts suggest that the shared identity described in Theme 1 was enacted in how participants organized demonstrations and cabildos and related to one another. We interpret this as an example of collective self‐objectification (CSO), through which the meanings and values defining an emergent *we* were realized in concrete social arrangements (Drury & Reicher, 2009).

### Theme 3: Alternatives that felt feasible and desirable

This theme explores how the forms of organizing and relating described in Theme 2 shaped what interviewees understood as possible and desirable. Their experience of participatory and community‐based practices across the uprising strengthened their sense that alternative social relations could work on a broader scale and were worth sustaining. We interpret this strengthened sense of their feasibility and desirability as empowering outcomes of collective self‐objectification.

#### From marginal ideals to workable practices

As described above, everyday life prior to the social explosion was widely experienced as depoliticized and politically stagnant, marked by resignation and a sense that meaningful alternatives were unrealistic or unattainable. In contrast, most interviewees described a marked shift in what felt socially and politically possible through the uprising: practices and relations that had seemed marginal, utopian or impractical began to appear more viable. As one participant explained:

For about two years, I'd been involved for real in groups applying those more community and participatory logics and all that. But what changed at that moment, during the uprising, was that I finally felt that these ways of doing things were possible… that they could really work (…) Before, I used to think that these new participatory practices would never catch on. (Paulina, Santiago)

Although she and others had prior experience with participatory or community‐based organizing, the interviewee indicated that these practices were experienced as difficult to sustain or extend beyond small‐scale contexts. The uprising disrupted this perception: what had seemed implausible came to feel achievable, largely through the discovery that others not only shared similar aspirations but also were willing to act on them. As the same interviewee explained when asked what made these alternative relations seem possible:

People were participating so much more in everything. Instead of criminalising protest, they were joining in. It was also intergenerational, and there was this sense of breaking away from your routine to be in those spaces. There was frequent talk of *us*, of *the people*, of a new Chile; and a shared sense of starting over that seemed to run through everyone who took part… (Paulina, Santiago)

This account illustrates how mass participation and visible public support provided collective validation that transformed personal ideals into shared, tangible possibilities. This heightened sense of feasibility also appeared to arise from directly experiencing forms of organizing grounded in shared values as workable in practice. For example:

Carolina I think that once we abandoned the idea of individualism and began practising collaboration, solidarity, and working together as a collective, it became powerful to see that it was working. Because it did work for three months!

Interviewer What made you feel that it worked?

Carolina When you saw people come to our assembly, and that people would come back…seeing everyone participate, that sense of cohesion that we developed through the social explosion (…) When you manage to come together and work for a common goal, it's hard to let go of each other afterwards. That's why we wrote that on a placard like I told you: “We took so long to find each other, that we now don't want to let go”.

(Carolina, Santiago)

Here, a greater sense of the feasibility of alternatives was tied to the effective functioning of collective life itself: cooperation held despite differences, assemblies sustained momentum and people kept returning. Against a backdrop of isolation and disillusionment, sustained participation provided tangible evidence that collective ways of organizing were viable. In this way, forms of organizing and relating on their terms no longer seemed like wishful thinking; they had been lived and tested in the streets and *cabildos* of Chile.

#### Experiencing alternative relations as worth sustaining

As described above, everyday social relations prior to the social explosion were widely experienced as alienating and unjust. The following extract captures how, for many, the social explosion brought experiences that radically contrasted with—and at times overturned—these alienating relations:

The exchanges in the different self‐convened spaces [of the uprising] were really beautiful… it felt like a breath of fresh air – realising that you weren't alone, hating everything, hating Chilean society so individualistic, so indifferent to social issues (…) It was a moment when you thought “at last”. “At last we stopped staying silent”. And it was so beautiful to be able to share with so many people, to build those bonds, to sit in the square talking about politics, making decisions, discussing the kind of Chile we wanted (…) In those encounters, you could dream – and even if those dreams were hard to reach, just saying them out loud with others, and realising that others wanted similar things, brought your soul back to life… (Paloma, Santiago)

As this account illustrates, by organizing collectively and shaping their spaces of resistance on their own terms in demonstrations, cabildos and neighbourhood assemblies, interviewees experienced forms of social relations that felt revitalizing and restorative. Across interviews, most participants described discovering in resistance spaces a sense of community and vitality that had been missing, and recognizing these as worth sustaining. In this way, dialogue, shared decision‐making and mutual support not only served as enabling conditions for resistance but also became meaningful in themselves, as cooperation, trust and solidarity were recognized as desirable bases for collective life. As one explained:

When those opportunities open up and you experience that civic energy: that energy from your neighbour, the one you'd never spoken to before, who's now out in the street meeting you and offering you a glass of water, for example… right away you think, “this is the Chile that we want” (…) A Chile where there is social fabric, where there's community, where you can rely on one another's support, where you're not afraid to talk to your neighbour, where you move beyond individualism (…) I think that, deep down, every human being is social and needs others. (Andrea, Santiago)

Here, in contrast to the fragmentation of everyday Chile, the uprising is portrayed as providing a glimpse of alternative ways of living together that now felt both possible and worth sustaining.

### Theme 4: Prefigurative realization: Living the change they sought

This theme explores how most interviewees came to understand aspects of their participation as doing more than opposing the existing order: through the uprising, they felt they were already living elements of the society they sought. These understandings drew on the experiences described in Themes 2 and 3, which gave political commitments shaped by prior activism and community organizing renewed practical and emotional force. They were articulated in two overlapping ways: through an emphasis on aligning means and ends (‘be the change you want to see’) and through a sense of ‘living the future through the uprising’.

The first of these was evident in how interviewees reflected on the organization of resistance itself. Rather than viewing it as primarily strategic—where principles are often subordinated to tactical goals—many came to see the importance of organizing practices in ways consistent with the kind of society they wanted to build. For example:

Leandro [before the uprising] I was drawn to horizontality as something tied to anarchism… the idea of a horizontal society, without racism, without prejudice, without hierarchies. But there [in the social explosion] I learned that you didn't need to have read anarchism to like anarchist ideas. Many people, I think, would be attracted to anarchist ideas but never realize it, because they don't get to live them. Because those who hold power in society, especially the media and politicians, make us afraid of political ideas that challenge the status quo.

Interviewer How did you learn that?

Leandro We were living horizontality and learning that it was common sense… very beautiful, but still common sense… learning from that encounter with one another, learning that if what you want is an egalitarian society, how can I impose my own voice over someone else's, you know? And at the same time, I can't let someone else impose their voice over me, you know?

(Leandro, Santiago)

In this account, the interviewee reasons that the egalitarian society he aspired to required organizing and acting together in ways that reflected it: treating one another as equals, refusing imposed voices and making decisions collectively. While many were already sympathetic to egalitarian and participatory ideals, it was through collective experimentation in the uprising that these principles became concrete and widely shared: ‘common sense’ ways of relating and organizing social and political life.

Other accounts framed the uprising as already embodying a desired future society:

When things explode and you see so many people with similar beliefs about the world as you, you feel alive! (…) I feel that what we lived through – not only when protesting, but the part where you were, for example, talking to people on the bus [when the metro was shut down], and not everyone was glued to their phones, or people offered to take you home, or things like that – was already like a new country. I mean, like we've already experienced it, in a way. It wasn't institutionalised, it wasn't channelled in some way… but at the grassroots level, we already lived it. (…) It's as if there are moments where time opens up, where there is like a social effervescence, that tells you “yes… life can be different”. (…) I think these things [time gaps] are very practical; you have to live them yourself. (Marcela, Santiago)

Here, the participant depicts the uprising as a moment when the future was not only imagined but also *lived*. The suspension of routine and the emergence of mutual care, cooperation and community made tangible a form of social life that contrasted with the isolation of everyday Chile. These practices were not experienced as merely incidental or strategic but as feasible and fulfilling ways of living together. Like others, she came into the uprising with beliefs about social change shaped by earlier organizing experiences, but it was through collective action—and witnessing these beliefs enacted by others—that they gained validation and emotional force. In the functioning of *cabildos*, street protest and everyday gestures of care, many interviewees experienced a sense that the society they aspired to was not merely being fought for, but was already, in some measure, being lived in the present.

### Strategic and outcome‐oriented accounts

Not all interviewees described their participation in prefigurative terms. Three interviewees identified with the aims of the mobilization and took part in the same practices others found empowering, but did not appear to interpret them as empowering examples of collective enactment or as validating alternative ways of organizing social life. Instead, their accounts centred on strategic effectiveness, organizational shortcomings and the translation of collective action into institutional outcomes.

In two interviews, this orientation was articulated through concerns about how collective action could produce concrete political outcomes:

The *cabildos* were places where people could talk about what was going on and draw energy from the discontent (…) But they were very challenging, because the issue was about how to give political direction to the discussions without imposing a position (…) For me, the challenge was always how to think about whether those discussions could lead to something concrete (…) constitutional change, legal reforms, or public policies. (…) rather than just staying at the level of discussion. (Andres, Santiago)

Here, *cabildos* are recognized as valuable spaces for articulating grievances and sustaining mobilization, but are assessed primarily in terms of whether discussion could be translated into institutional change. Their importance, therefore, lies in what they might produce beyond the immediate experience of participation.

A further interviewee assessed *cabildos* primarily through what she saw as their organizational limitations:

Interviewer What were you trying to do in the cabildos?

Pilar What we all wanted was collective reflection, some leadership, a discourse (…) To give direction to that anarchy of demands (…) because there was no discourse, there was no leader (…) In the 1980s, things were clearer… there were clear leaders, there was a discourse… there was direction.

(Pilar, Rancagua)

Here, *cabildos* are assessed in terms of what they lacked—direction, leadership, a coherent discourse—rather than through the relations enacted within them. While this interviewee elsewhere endorsed horizontal and autonomous organizing, her account reflects the difficulty of reconciling these practices with more traditional expectations of effective political organization.

Taken together, these accounts suggest that participation in similar practices did not necessarily carry the same political or psychological meaning. Whereas other participants described them as enactments of collective identity that made alternative relations appear feasible and desirable, these interviewees evaluated them primarily as means of producing political outcomes beyond the immediate experience of participation.

## DISCUSSION

The present study examined how participants in the 2019 Chilean social explosion made sense of the forms of organizing and relating that unfolded through the mobilization. It explored whether participants understood these experiences in prefigurative terms and, if so, how such understandings took shape in a context shaped by prior political organizing experience.

Across interviews, most participants described aspects of their involvement in ways that reflected a prefigurative understanding of participation. Although all had prior experience in activism or community organizing, these understandings did not appear merely to reflect pre‐existing social change commitments. Instead, participants described how, through the uprising, a shared sense of collective identity—organized around values of autonomy, dignity and a collective desire for change—took shape and was enacted through resistance practices. Through this process of collective self‐objectification (Drury & Reicher, 2009), values associated with the uprising were materialized in demonstrations, *cabildos*, and everyday relations among participants. As this identity was enacted, many experienced collective empowerment, as forms of collective life organized around these values came to feel feasible, compelling and widely shared. Together, these experiences shaped how many interviewees understood their participation: not only as a means to an end but also as a partial enactment of the social relations they sought. We describe this possible shift in the meaning assigned to participation as *prefigurative realization*. In participants' accounts, this realization appeared to take shape cumulatively as they made sense of their organizing and relating across the uprising as a whole, rather than arising in a single moment of revelation.

At the same time, three interviewees narrated their participation primarily through strategic or outcome‐oriented frameworks, locating its political significance beyond immediate collective practices. Although they engaged in many of the same forms of organizing as the other interviewees, they did not retrospectively understand these experiences as politically generative in themselves; instead, they judged their political significance by the extent to which they led to formal political outcomes or coherent political direction or leadership. Together, these cases reinforce the argument that prefigurative meaning lay not in the practices alone, but in how participants understood the organization and relations within the struggle: as part of the political change being pursued, rather than primarily as instruments for achieving outcomes elsewhere.

The present findings contribute to a small but growing body of psychological research on prefigurative politics in collective action (e.g. Acar & Uluğ, 2016; Aguilera & Drury, 2025; Azamian & Dittmer, 2025; Clarke & Drury, 2025; Mao et al., 2021; Vestergren et al., 2025). Recent work has begun to move beyond treating prefiguration as a given ideological orientation, highlighting the role of the lived experience of struggle in shaping how collective action comes to be understood as prefigurative enactment (Acar & Uluğ, 2016; Aguilera & Drury, 2025).

Previous ESIM‐informed research on the 2018 Nicaraguan uprising, for example, documented how prefigurative understandings emerged among participants with limited prior activist experience, as new forms of organizing were enacted and recognized as effective and fulfilling ways of structuring collective life (Aguilera & Drury, 2025). The Chilean case offers a contrast, as it was shaped by earlier cycles of mobilization and more established repertoires rooted in horizontal and participatory traditions. Here, prefigurative understandings were less a matter of discovering alternative social relations than of seeing existing values and practices enacted by a much wider collective. The findings suggest that collective self‐objectification validated and sometimes reconfigured existing commitments through their enactment at scale.

Acting together at scale and shaping the internal structure of the movement in line with shared values made these ways of organizing feel more widely shared, practically viable and politically compelling. This suggests that even in contexts shaped by established activist histories, prefigurative understandings can develop through the relation between prior experience and the unfolding dynamics of struggle, as political meanings are validated, reworked and given renewed practical and emotional force through practice.

### The Chilean social explosion and the psychology of collective action

Beyond the specific contributions to debates on prefigurative politics, our study speaks to broader questions about how collective action is understood in social psychology. We situate these findings in relation to mainstream research, focusing on how collective action is conceptualized and lived in contemporary mobilizations.

Much social–psychological research adopts a predictor‐outcome architecture in order to explain when and why individuals participate in collective action. Within this framework, participation is treated as a behavioural outcome to be explained, typically operationalized through individual, institution‐facing acts, such as attending a demonstration, signing a petition or supporting a campaign. Attention therefore centres on psychological antecedents, including given variables such as social identification, perceived injustice, efficacy and moral conviction (Agostini & van Zomeren, 2021; van Zomeren et al., 2008).

This approach has generated valuable insights into why people engage in collective action across contexts and has helped establish the topic as a key concern within social psychology. Yet this predictor‐outcome architecture rests on assumptions about what collective action is and where its psychological significance lies. By centring analysis on antecedent states, this approach locates the key psychological moment prior to the action itself. Moreover, because participation is treated as the outcome to be explained, social change tends to be located after or beyond the action itself, in expected external outcomes. As a consequence, what takes place *within* collective action—how people organize, relate and experience themselves and others through participation—tends to appear, if at all, as contextual background rather than as a central object of inquiry.

The experiences described by participants in the Chilean case highlight the significance of this omission. While participants did reflect on why they joined or what the movement might achieve, they consistently emphasized what unfolded through acting together. Practices such as self‐organized demonstrations, neighbourhood assemblies and shared decision‐making were described not merely as tactics but as integral to how participation itself was experienced and understood.

Importantly, these experiences were not simply expressions of pre‐existing political beliefs. While our interviewees entered the uprising with prior activist histories and social change beliefs, the uprising provided a context in which these beliefs were enacted collectively and reworked through practice. Acting together at scale enabled many participants to experience collective organizing on their own terms as both possible and effective, reshaping how they understood themselves, their actions and the possibilities of change.

When we move beyond some of the assumptions built into antecedent‐driven approaches, an alternative understanding of collective action comes into view—one that resonates with our interviewees' accounts and with broader shifts in contemporary mobilizations. This perspective does not limit the psychological significance of mobilization primarily to the states or beliefs that precede action, nor does it restrict collective action to discrete behaviours directed towards institutional outcomes. Instead, it centres the dynamics that can emerge through participation itself. Thus, collective action appears not only as a behavioural outcome but also as a potentially transformative social practice through which relations, capacities and political meanings can be produced or reshaped (cf. Drury & Reicher, 2009; Vestergren et al., 2025).

Such a practice‐based understanding is central to ESIM, which explains how participation transforms collective identity. By focusing on the outcomes of enacting these identities, our findings extend ESIM from an account of identity transformation to an explanation of how collective action comes to function as a prefigurative practice, in which the struggle is not only oriented towards social change but constitutes its partial enactment.

This reorientation draws attention to dynamics often missed by mainstream antecedent models and, in doing so, suggests shifts in how collective action and its relationship to social change are understood. First, it shifts the primary object of analysis from antecedent states to collective practices. Rather than treating participation as the endpoint of psychological explanation, this perspective foregrounds collective action as a site in which meanings, identities and relations can take shape. Second, it unsettles the field's reliance on linear explanatory models, pointing towards recursive accounts in which participation and psychological dynamics are mutually constitutive (cf. Bou Zeineddine & Leach, 2021; Drury & Reicher, 2000, 2009). From this standpoint, collective action is not simply the expression of pre‐existing psychological states but a generative process that can feed back into participants' understanding of themselves, others and social change, with implications for future actions and for their everyday lives (Drury & Vestergren, 2025).

Finally, approaching collective action as a potentially transformative practice has wider implications for how its relationship to social change is understood. Rather than treating collective action only as a vehicle for later institutional change, this perspective emphasizes the need for frameworks capable of analysing forms of political action in which social change may be underway through participation itself—namely in the organization of social relations, the reconfiguration of power and agency and the lived experience of struggle.

The need for such a shift becomes particularly salient in contexts characterized by institutional distrust, inertia or political exclusion, where groups lack confidence that existing political structures will organize collective life on favourable terms or respond meaningfully to their demands. In these settings, participants are often required to organize the internal life of their movements themselves, developing novel forms of coordination, decision‐making and mutual support that break from everyday hierarchies and relations of power. In this context, structuring collective action around alternative shared values can be empowering, insofar as it reveals the contingency of the existing order, counters everyday experiences of powerlessness and renders alternative social relations feasible and newly compelling as forms of collective life.

### Limitations and future research

The limitations of the present study also point towards important directions for future research. The analysis relies on retrospective interview accounts collected approximately 5 years after the peak of the 2019 mobilization. As a result, the study could not directly examine how participants made sense of their practices across different phases of the uprising or how these meanings developed over time. The analysis therefore examines participants' later reconstructions of the significance of their actions rather than how these understandings emerged, shifted or were contested during the mobilization itself. Nor can it establish how widespread these understandings were across the wider mobilization.

Longitudinal designs would allow closer examination of how participants come to understand collective practices in prefigurative terms and how these understandings develop, stabilize or weaken over time. However, mass uprisings are difficult to study longitudinally, both because of their unpredictability and because of the practical and ethical constraints they present for sustained research engagement. Future research might therefore examine non‐insurrectionary contexts—such as protest encampments, community initiatives or environmental struggles—where longer term study is more feasible. Such contexts would allow researchers to follow collective practices closely over time and examine when and how they come to be understood in prefigurative terms under different conditions.

## CONCLUSION

This study examined how participants in the 2019 Chilean social explosion made sense of their involvement in demonstrations and *cabildos* during the mobilization. Building on ESIM and in‐depth interviews, we explored whether they understood aspects of these experiences in prefigurative terms and how such understandings related to prior activist commitments and to processes that emerged through participation. Across accounts, most participants described aspects of their involvement as enacting desired social relations. Although all had prior political experience, our analysis suggests that these understandings were not simply given in advance but also took shape through collective action, as shared values were enacted in ways that made alternative relations feel increasingly viable, compelling and widely shared.

We argue that these findings make the case for social psychology to broaden its focus in collective action research—to move beyond explaining why people act and attend more closely to what becomes possible through participation. In this light, prefigurative politics invites a rethinking of how social change is conceptualized: not just as a distant goal, but as something that can begin to take shape through collective practice itself.

## AUTHOR CONTRIBUTIONS

**David Clarke:** Conceptualization; methodology; investigation; formal analysis; writing – original draft; writing – review and editing. **John Drury:** Writing – review and editing; conceptualization; methodology; supervision.

## FUNDING STATEMENT

No funding.

## CONFLICT OF INTEREST STATEMENT

None of the authors have a conflict of interest to disclose.

## ETHICS STATEMENT

This study received ethical approval from the University of Sussex Research Ethics Committee (Ref: ER/DAC29/7).

## Supporting information


**Supplementary Material** bjso70133‐sup‐0001‐Supinfo.pdf

## Data Availability

Anonymised interview extracts supporting the findings of this study will be made available on the Open Science Framework upon publication.

## Footnotes

^1^
Following common Chilean public and scholarly usage, we retain *estallido social* and translate it as “social explosion” (e.g. Gordon‐Zolov, 2023; Heiss, 2021; Woods, 2022). Other labels in the literature include “social outburst”, “social unrest” and “social uprising”.
