# Supplementary material for: Enacting desired change in the present: Explaining prefigurative collective action during Chile's 2019 social explosion
Source: Br J Soc Psychol. 2026 Sep 24;65(4):e70133. doi: 10.1111/bjso.70133 (PMC13612935; doi:10.1111/bjso.70133)
Supplement: Supplementary file 1 [file BJSO-65-0-s001.pdf]

Interview schedule

i) Preamble

This interview is part of a research project about recent Latin American uprisings. The aim of the interview is to explore your experiences of participation in the Chilean 2019 "Social explosion". Let me remind you that everything you share will remain confidential and that you are free to stop the interview at any time.

ii) Introduction

- Can you tell me a bit about yourself?
    - How old are you?
- What was your life like before the uprising?
    - Did you have a job? Were you a student?
- How did you view national Chilean politics before the protests began?
    - How did you view the government? How did you view the opposition parties?
- Had you been involved in any protest before?

iii) Exploring prefigurative practices bottom-up

Participation in popular assemblies

- Can you tell me a bit about your participation in the popular assemblies?
    - How did you get involved?
- What was the role of the popular assemblies?
    - What were you trying to achieve?
- Was this political experience different from your previous experience with politics? (e.g., elections, traditional parties)
- Did these spaces allow you to practice different values?
    - In what ways? What values?
- Was this experience inspiring?

If participants mention prefigurative practices (repeat for each practice)

- Why did you adopt this practice?
- Was it effective? Did it work well?
- How did you feel about what you were doing?
    - Did your feelings or views about it change over time?
- Did everyone in the group adopt it and coordinate?
- Did discussions with other members adopt it and coordinate?
- Were there any problems in doing it?
- What did you think afterwards about such spontaneous forms of organization? Good or bad?
- Did it make new forms of organizing seem more feasible?
- Did it make a new society seem more feasible?

iv) General participation in the "Social explosion" and changing social psychological processes.

State repression and protesters' self-conceptions.

Before the protests

- Can you tell me about how you felt about the police prior to being involved in the protests?

    - And about the army?

During their participation

- What were your attitudes to the government following police and army violence against protesters?
    - How did you view the police/army now?
- Did police/army violence change what forms of protest you felt were justified?
- As protests developed, were you protesting the metro fare rise? Or state repression? Or something else?

Relationship with other protesters / assembly members

- How did you feel about other protesters during your experience of the initial protests?
- How did you feel about them as protests developed? And as protests grew nationwide?
- How did you feel about your fellow assembly members? Did these feelings change as the movement grew?
- Was there public support for the Social explosion? How did that influence your participation?

Empowerment processes and reflection on general participation

- How did your sense of what the movement could achieve change as the protest developed?
    - What influenced these changes?
    - How did those changes make you feel?
- Can you tell me about any moments when you felt a sense of victory? Or where you felt inspired?
- How did your participation in the Social explosion or assemblies affect your sense of what national politics might be like?
    - In what way was this different to the recent political context?
- How did your experience in the Social explosion/assemblies influence your views of the existing constitution and the possibility of a new one?

v) Closing

- Is there anything else you would like to say about your experiences?
- Do you know anyone else who would like to participate in the study?
